# Endocrine therapy considerations in postmenopausal patients with hormone receptor positive, human epidermal growth factor receptor type 2 negative advanced breast cancers

**DOI:** 10.1186/s12916-015-0280-0

**Published:** 2015-03-05

**Authors:** Ilenia Migliaccio, Luca Malorni, Christopher D Hart, Cristina Guarducci, Angelo Di Leo

**Affiliations:** Translational Research Unit, Hospital of Prato, Istituto Toscano Tumori, Via Suor Niccolina 20, 59100 Prato, Italy; “Sandro Pitigliani” Medical Oncology Department, Hospital of Prato, Istituto Toscano Tumori, Via Suor Niccolina 20, 59100 Prato, Italy

**Keywords:** Endocrine therapy, HER2 negative breast cancer, Hormone receptor positive breast cancer, Metastatic breast cancer

## Abstract

The standard of care for patients with hormone receptor positive, human epidermal growth factor receptor type 2 negative advanced breast cancer is endocrine therapy. Endocrine agents, including aromatase inhibitors, tamoxifen, and fulvestrant, are often administered alone as first line treatment and demonstrate durable responses with limited side effects. Endocrine resistance represents a major clinical problem. In the future, poly-endocrine therapy and combination therapies with biological agents might become valuable options for the first line treatment of hormone receptor-positive advanced breast cancer. However, it will be critical to develop clinical tools that can reliably identify the subgroup of patients most likely to benefit from endocrine therapy alone, and those who might benefit from alternative approaches.

Herein, we will review and discuss current issues in the endocrine treatment of postmenopausal patients with hormone receptor positive, human epidermal growth factor receptor type 2 negative advanced breast cancer.

## Introduction

Postmenopausal patients with hormone receptor positive (HR+), human epidermal growth factor receptor type 2 negative (HER2–) tumors represent the majority of patients with advanced breast cancer (ABC). Despite the sometimes indolent course of the disease, HR+ HER2– ABC remains incurable. Current goals of therapy are therefore to prolong survival and palliate symptoms while maintaining a good quality of life. In the majority of women with HR+ HER2– ABC, these goals can be achieved with endocrine therapy, which represents the standard of care for first line treatment [1]. Available agents for postmenopausal patients include steroidal (exemestane) and non-steroidal (anastrozole and letrozole) aromatase inhibitors (AIs), selective estrogen receptor modulators (tamoxifen or toremifene), and the estrogen receptor (ER) down-regulator fulvestrant. These agents are generally effective and well tolerated, but not all patients benefit from them equally [1].

At present, due to the lack of predictive biomarkers that can identify subgroups of patients that will derive the largest benefit from endocrine therapies, treatment decisions regarding the choice between endocrine therapy and chemotherapy are based on clinical criteria such as disease-free interval, extent of visceral metastatic involvement, and degree of symptoms. When clinical criteria support its use, endocrine therapy is typically administered alone, but future options might include poly-endocrine therapy or combination with new biological agents.

## Endocrine therapy alone

Tamoxifen has long been established as an effective first line treatment of postmenopausal women with HR+ ABC [2]. Phase III trials then compared AIs or fulvestrant to tamoxifen in such patients demonstrating equivalent [3-10] or superior efficacy to tamoxifen [5,7,10], being equally well tolerated, and AIs have become the standard of care in first line. More recent data suggests that fulvestrant may be superior to AIs in this setting. The phase II randomized Fulvestrant fIRst-line STudy (FIRST) trial compared fulvestrant high dose (500 mg) to anastrozole for first line treatment of postmenopausal women with HR+ ABC. Clinical benefit rate, the primary study endpoint was similar for the two groups [3]. However, at the time of a more mature follow-up, median time to progression (TTP) was significantly longer for the patients receiving fulvestrant (23.4 vs. 13.1 months; hazard ratio (HR), 0.66; 95% CI, 0.47–0.92; *P* = 0.01) [11]. Updated results, recently presented, showed that also median overall survival (OS) was significantly longer for fulvestrant (54.1 months) versus anastrozole (48.4 months; HR, 0.70; 95% CI, 0.50–0.98; *P* = 0.041) [12]. These results require confirmation in a phase III trial, which is currently underway.

Overall, these trials demonstrated that, in a proportion of women undergoing first line single agent endocrine treatment, disease remissions lasting more than 12 months can be observed. Indeed, in the FIRST trial the median TTP for anastrozole was 13.1 months [11]. In two trials comparing anastrozole versus tamoxifen, median TTP for anastrozole was 11.1 months [7] and 8.2 months [6]. However, a high proportion of patients enrolled in these trials had not received prior adjuvant endocrine therapy. Whether the same results would be achieved in patients who received adjuvant endocrine therapy (commonly with an AI) remains an open question.

Current clinical tools to distinguish patients who will benefit from endocrine therapy alone from those who will require an alternative approach are sub-optimal. Hierarchical cluster analysis has revealed that HR+ tumors can be divided into at least two molecular subgroups, namely luminal A and luminal B [13,14], with distinct clinical behavior and response to chemotherapy and endocrine therapy [15]. Additionally, in the adjuvant setting, molecular signatures are emerging as a powerful tool that could aid clinicians in therapeutic decision [16]. However, the clinical utility of molecular subtypes and signatures in patients with HR+ HER2– ABC is yet to be established. The aforementioned clinical trials did not collect tumor samples, thus subgroup analyses based on molecular subtypes or any other biomarker could not be made.

## Poly-endocrine therapy

Endocrine agents have different mechanisms of action; therefore, drug combination strategies of different endocrine agents might be an approach to improve the effectiveness of endocrine therapy. Indeed, some preclinical data support poly-endocrine therapy strategies [17], but results from clinical trials conducted in the metastatic setting are contradictory [18,19].

The Fulvestrant and Anastrozole Combination Therapy (FACT) trial is a randomized phase III study comparing fulvestrant low dose (250 mg) and anastrozole in combination versus anastrozole alone as first line treatment of postmenopausal women, or premenopausal women receiving a gonadotropin-releasing hormone agonist, with HR+ ABC. It enrolled 514 patients and the primary end point was TTP. TTP, OS, clinical benefit, and objective response rates were neither statistically nor clinically different between the two treatment groups (HR for TTP, 0.99; 95% CI, 0.81–1.20, *P* = 0.91; HR for OS, 1.0; 95% CI, 0.76–1.32, *P* = 1.00) in this trial [18].

Another phase III trial, designed and conducted by the Southwest Oncology Group (SWOG) Cooperative Group, randomly assigned 694 postmenopausal women with HR+ ABC to receive either anastrozole (group 1), or anastrozole and fulvestrant low dose in combination (group 2) as first line treatment. The median progression free survival (PFS) was 13.5 months in group 1 and 15.0 months in group 2 (HR, 0.80; 95% CI, 0.68–0.94; *P* = 0.007). OS was also longer in group 2 (47.7 months vs. 41.3 months in group 1; HR, 0.81; 95% CI, 0.65–1.00; *P* = 0.05) [19].

The three-arm, phase III SoFEA trial randomized postmenopausal patients with HR+ ABC to receive fulvestrant low dose plus anastrozole (n = 243), fulvestrant low dose plus placebo (n = 231), or exemestane alone (n = 249). It differed from the FACT and SWOG trials by analyzing a population that had progressed on non-steroidal AI. No significant difference in PFS, the primary endpoint, was seen between the three groups (fulvestrant plus anastrozole vs. fulvestrant plus placebo: HR, 1.00; 95% CI, 0.83–1.21; *P* = 0.98; fulvestrant plus placebo vs. exemestane: HR, 0.95; 95% CI, 0.79–1.14; *P* = 0.56), nor was an OS difference seen [20].

It must be highlighted that, in all these trials, the fulvestrant dose (250 mg) and schedule were chosen according to the standard in use at the time of studies design. The phase III Comparison of Faslodex in Recurrent or Metastatic Breast Cancer (CONFIRM) trial demonstrated that a higher fulvestrant dosing schedule (500 mg) was superior to the low dose in terms of PFS and OS [21,22]. The suboptimal dose of fulvestrant used in these trials might have therefore influenced the results.

These trials did not collect tumor samples, and analyses of treatment by luminal subtype or by other tumor-specific biologic factor were not made, but an unplanned subgroup analysis of patients enrolled in the SWOG trial suggested that the majority of benefit seen was in patients previously untreated with tamoxifen. The median PFS among women untreated with tamoxifen was 12.6 months in group 1 versus 17.0 months in group 2 (HR, 0.74; 95% CI, 0.59–0.92; *P* = 0.006), while among women previously treated with tamoxifen, the estimated median PFS was 14.1 months and 13.5 months, respectively (HR, 0.89; 95% CI, 0.69–1.15; *P* = 0.37). The interaction between treatment and use of prior adjuvant tamoxifen therapy was not significant (*P* = 0.22) [19]. In the FACT trial, subgroup analysis did not suggest any interaction between previous exposure to endocrine therapy and treatment activity [18]. However, it included a substantially lower number of tamoxifen-naïve patients than the SWOG trial (171 vs. 414 patients, respectively) [18,19]. In the SoFEA trial, this subgroup analysis was not made, but patients with tumors with both ER and progesterone receptor (PR) positivity, favoring a luminal A, more endocrine-sensitive phenotype, seemed to derive greater benefit from the combination therapy.

Based on these data, it might be hypothesized that patients unexposed to prior endocrine therapy and with highly endocrine-sensitive tumors could derive the largest benefit from the combination of an AI and fulvestrant. However, in view of the contradictory results of the trials, it seems appropriate to wait for further evidence before considering the combination of AIs and fulvestrant as standard of care.

## Endocrine therapy in combination with biological agents

Some patients with HR+ ABC show primary resistance to endocrine therapy, and in the remainder, secondary resistance ultimately develops, representing a major clinical problem. The biology of resistance to endocrine therapy is complex and still not completely elucidated [23]. Preclinical evidence suggests that targeting the phosphatidylinositol 3-kinase (PI3K)-Akt-mammalian target of rapamycin (mTOR) [24] or the cyclin D1-Cyclin-Dependent Kinases 4 and 6 (CDK4/6) pathway [25] might increase endocrine sensitivity. Based on this rationale, randomized clinical trials have recently investigated whether combination therapies with biological agents targeting these pathways would improve PFS or OS of patients with HR+ ABC [26,27].

The phase III BOLERO-2 trial randomized 724 postmenopausal patients with HR+ HER2– ABC to receive everolimus, an mTOR inhibitor, and exemestane versus exemestane and placebo. The primary end point, PFS, was shown to be significantly improved in patients receiving everolimus compared to those receiving placebo according to both local (6.9 vs. 2.8 months; HR, 0.43; 95% CI, 0.35–0.54; *P* <0.001) and central assessment (10.6 vs. 4.1 months; HR, 0.36; 95% CI, 0.27–0.47; *P* <0.001) [26]. However, the combination did not confer a statistically significant improvement in OS (median OS: 31.0 months in the everolimus plus exemestane arm vs. 26.6 months in the exemestane plus placebo arm; HR, 0.89; 95% CI, 0.73–1.10; *P* = 0.1426) [28]. Given the remarkable PFS results, everolimus was approved by the Food and Drug Administration for the treatment of postmenopausal women with HR+ HER2– ABC in combination with exemestane, after failure of treatment with letrozole or anastrozole. However, the toxicity profile of everolimus is far from ideal. Serious adverse events were higher in patients receiving everolimus compared to those receiving placebo (55% and 33%, respectively) and a higher proportion of patients discontinued everolimus because of adverse events compared to placebo (29% vs. 5%) [28]. For this reason there is a great interest in identifying biomarkers of response to screen patients who are likely to derive the largest benefit from this compound.

mTOR exists in two different complexes, mTORC1 and mTORC2. Everolimus targets mTORC1, which signals via two major substrates, the p70 ribosomal protein S6-kinase (pS6) and the eukaryotic initiation factor 4E binding protein 1 (4EBP1) [24]. The activity of mTORC1 is regulated by the serine/threonine kinase Akt, a downstream effector of PI3-kinases. Activating mutations in the catalytic subunit of PI3-kinase (PIK3CA) occur in around 40% of ER+ breast cancers [29], but the hypothesis that PIK3CA-mutated breast cancers would derive the largest benefit from mTOR inhibitors was not confirmed in an exploratory analysis of the BOLERO-2 trial, although only a fraction of enrolled patients were included [30]. One explanation for these results might be that mutational status of PIK3CA does not correlate with pathway activation. A seminal work of Loi et al. indeed demonstrated that, in ER+ HER2− breast cancer, PIK3CA mutation surprisingly did not always result in elevated downstream signaling, and correlated with relatively low mTORC1 signaling [31,32]. Reverse phase protein array data from the The Cancer Genome Atlas also confirmed that phospho-AKT, phospho-pS6, and phospho-4EBP1, markers of PI3K pathway activation, were not necessarily elevated in PIK3CA mutated luminal A breast cancer [33]. These data suggest that mutational status of PIK3CA should be combined with assessment of downstream pathway activity to have a better prediction of everolimus benefit. In support of this, Loi et al. [32] showed, in a dataset derived from patients enrolled in a randomized, double blind, phase II neoadjuvant trial, that lower scores of a genomic signature of PIK3CA mutation (PIK3CA-GS) were able to identify those patients with the largest relative decreases in Ki67 (a surrogate marker of response) to letrozole/everolimus (R = −0.43, *P* = 0.008) compared with letrozole/placebo (R = 0.07, *P* = 0.58; interaction test *P* = 0.02). However, in a second dataset from pre-surgical patients using everolimus alone, the PIK3CA-GS was not correlated with relative change in Ki67 (R = −0.11, *P* = 0.37). In both datasets, changes in percentage of Ki67 decrease were not statistically different between PIK3CA mutant and wild-type breast cancer [32]. Additionally, translational studies within the TAMRAD trial, a multicenter phase II trial in which postmenopausal women with HR+ HER2– ABC previously treated with AI were randomly allocated to receive tamoxifen plus everolimus (n = 54) or tamoxifen alone (n = 57), have recently shown that the subgroups most likely to have an improvement in TTP with tamoxifen plus everolimus therapy compared with tamoxifen alone were patients with molecular evidence of PI3K pathway activation (i.e., high phospho-4EBP1, low 4EBP1) [34].

Another important point is that in the BOLERO-2 trial, PIK3CA mutational status was assessed mainly on primary tumor tissues [30]. Studies indicate that discordance in PIK3CA mutational status between primary tumors and metastases might occur [35-37], suggesting that we should reassess molecular pathway alterations prior to starting targeted treatment, either through analysis of metastatic tissue or, potentially, ‘liquid biopsies’. Indeed, the feasibility of assessing PIK3CA mutation in circulating tumor cells [38-41] and circulating free DNA [42-44] has already been demonstrated.

To further assess the role of PIK3CA mutations in breast cancer, a mutational analysis of PIK3CA/AKT1 and RAS/RAF was performed on 4,294 primary tumor samples from postmenopausal patients with ER+ breast cancer who had been enrolled in the Tamoxifen Exemestane Adjuvant Multinational phase III trial [29]. This study showed that PIK3CA mutations were more frequent in low-risk luminal breast cancer and were associated with significantly improved 5-year distant relapse-free survival in univariate analysis (HR, 0.76; 95% CI, 0.63–0.91; *P* = 0.003) [29]. These results are consistent with previous findings that PIK3CA mutations are more frequently seen in luminal A primary tumors and are associated with increased sensitivity to endocrine therapy [31,33,45]. Mayer and Arteaga suggested, in an accompanying editorial [46], that the apparent discrepant role of PIK3CA mutations in early versus late ER+ breast cancer might be explained by a predominant role of PIK3CA mutations in secondary endocrine resistance [46]. Data supporting this hypothesis come from preclinical studies demonstrating an increased pathway activation in long-term estrogen-deprived breast cancer cell lines [47] and from an exploratory subgroup analysis within the TAMRAD trial, which suggested that the everolimus benefit was seen more in patients with secondary hormone resistance than those with primary resistance [48].

In the absence of available biomarkers, clinical considerations regarding the population of the BOLERO-2 trial should be taken into account when selecting patients for combination therapy with everolimus. First, 84% of patients enrolled in the BOLERO-2 trial were sensitive to prior endocrine therapy. Endocrine sensitivity was defined as at least 24 months of endocrine therapy before recurrence in the adjuvant setting or a response or stabilization for at least 24 weeks of endocrine therapy for advanced disease [26]. Second, 84% of patients received everolimus as second line or more of therapy for advanced disease [26]. Accordingly, in our opinion, the combination of exemestane and everolimus could be considered an appropriate second line treatment option for patients who have demonstrated benefit from first-line endocrine therapy.

Several PI3K inhibitors are in clinical development for patients with HR+ HER2– ABC [49]. Recently, the results from the FERGI study, a phase II randomized trial of the PI3K inhibitor pictilisib plus fulvestrant versus fulvestrant plus placebo in patients with ER+ AI-resistant ABC, have been presented. These data showed that the addition of pictilisib to fulvestrant was associated with a non-statistically significant improvement in PFS for the combination versus the control arm (6.2 vs. 3.8 months; HR, 0.77; 95% CI, 0.50–1.19). The benefit from pictilisib was independent from PIK3CA mutational status, while the subgroup of patients that seemed to benefit more from the addition of pictilisib was that with ER+/PR+ tumors (PFS, 7.2 vs. 3.7 months in the combination and control arm respectively; HR, 0.46; 95% CI, 0.27–0.78) [50].

Clinical trials are currently investigating the safety and efficacy of three CDK4/6 inhibitors, palbociclib, abemaciclib, and LEE011, for the treatment of HR+ HER2– ABC [25]. The compound with more mature clinical results is palbociclib. The phase II, randomized PALOMA 1 trial was designed as a two-part study to evaluate palbociclib in combination with letrozole versus letrozole alone for first line treatment of postmenopausal patients with HR+ HER2– ABC. Part 1 of the study enrolled 66 unselected patients, while the Part 2 enrolled 99 patients with tumors positively screened for cyclin D1 amplification and/or loss of p16; the primary end point was PFS. The final analysis showed a statistically significant improvement in PFS for the combination arm versus letrozole arm (20.2 vs. 10.2 months; HR, 0.488; 95% CI, 0.319–0.748; one-sided *P* = 0.0004). When Part 1 and Part 2 were analyzed separately, treatment effects were maintained (Part 1: HR, 0.299; 95% CI, 0.156–0.572; one-sided *P* < 0.0001; Part 2: HR 0.508; 95% CI, 0.303, 0.853; one-sided *P* = 0.0046) [27]. Side effects were mainly hematological, with grade 3/4 neutropenia and leucopenia reported in 54% and 19% of patients receiving palbociclib, respectively [27]. At present, palbociclib is not registered for the treatment of breast cancer and confirmatory results from ongoing phase III trials are eagerly awaited.

Numerous biological agents are currently being investigated in combination with endocrine therapy for the treatment of postmenopausal patients with HR+ HER2– ABC, including histone deacetylase, Akt, and IGF receptor inhibitors [51,52]. For the future clinical development of these agents, understanding which subgroup of patients is more likely to benefit from the combination with endocrine therapy is of critical importance. This might be achieved i) with sub-group analyses correlating the clinical activity with the degree of response to prior endocrine therapies defined according to standard criteria and ii) with the identification of biomarkers of response.

## Conclusions

Endocrine therapy is the mainstay of first line treatment for postmenopausal women with HR+ HER2– ABC. Endocrine agents in mono-therapy demonstrated high efficacy and tolerability, but endocrine resistance commonly arises. Developing clinical tools able to reliably identify patients that will benefit from endocrine therapy alone and those that will require different approaches, such as poly-endocrine therapy or combination with biological agents, is an urgent clinical need. Studies investigating poly-endocrine therapy are contradictory and need further validation. Numerous new agents in combination with endocrine therapy are in clinical development for patients with HR+ HER2– ABC. However, when considering a combination therapy in such patients any additional benefit must be carefully weighed against additional toxicity and costs.

## Abbreviations

4EBP1: 4E Binding protein 1
ABC: Advanced breast cancer
AIs: Aromatase inhibitors
CI: Confidence interval
CONFIRM: Comparison of Faslodex in Recurrent or Metastatic Breast Cancer trial
ER: Estrogen receptor
FACT: Fulvestrant and Anastrozole Combination Therapy
FIRST: Fulvestrant fIRst-line STudy
HER2–: Human epidermal growth factor receptor type 2 negative
HR: Hazard ratio
HR+: Hormone receptor positive
mTOR: Mammalian target of rapamycin
OS: Overall survival
PFS: Progression free survival
PI3K: Phosphatidylinositol 3-kinase
PIK3CA: Catalytic subunit of PI3-kinase
PIK3CA-GS: Genomic signature of PIK3CA mutation
PR: Progesterone receptor
pS6: S6-kinase
SWOG: Southwest Oncology Group
TTP: Time to progression
